# Supplementary material for: An all-optical multidirectional mechano-sensor inspired by biologically mechano-sensitive hair sensilla
Source: Nat Commun. 2024 Apr 4;15:2906. doi: 10.1038/s41467-024-47299-0 (PMC10994919; doi:10.1038/s41467-024-47299-0)
Supplement: Supplementary file 1 — Supplementary Information [file 41467_2024_47299_MOESM1_ESM.pdf]

Supplementary Information for "An All-Optical Multidirectional Mechano-Sensor Inspired by Biologically Mechano-Sensitive Hair Sensilla"

Supplementary Note 1: Biological Systems of Mechano-Sensitive Hair-Like Sensilla

Mammals, including humans, rely on cochlear hair cells with stereocilia and kinocilia for the transduction of mechano/acoustic stimuli into membrane potential changes in receptor neurons[1,2], and some mammals (felid, murine, etc.) also use whiskers to provide nonvisual but acute sensory information, such as vibrations, under poor visibility conditions[3]. Moreover, the cilia of crickets detect mechanical signals in the surrounding environment and courtship and prey signals[4,5]; fishes use their lateral line system to detect water motions and pressures for predator and prey detection, object avoidance and social behaviors[6-8]; and spiders use the deflections of the tactile hair protruding from their exoskeleton's surface to detect forces and prey or predator signals propagated through airflow or wet areas[2,9].

Supplementary Note 2: Experimental Proof of the Force Sensing Mechanism

To analyze the main reason for the shift in the resonance wavelength, a solid microsphere-based mechano-sensor and a hollow microbubble-based mechano-sensor were prepared. Both mechano-sensors were packaged in the same polymer matrix (MY-133-V2000), and external forces (F_r, φ = 180°) were applied at the same axial position. As shown in Supplementary Fig. 1, the solid microsphere-based mechano-sensor has very little force sensitivity. In addition, microbubble-based mechano-sensors encapsulated with four different commercial polymer matrices (Supplementary Table 1) were prepared. To study the effect of the polymer matrix on the force sensitivity of the mechano-sensor, the geometric parameters of the microbubbles and the micro-hairs are the same in all four mechano-sensors, and the external forces (F_r, φ = 180°) were applied at the same axial position. As shown in Supplementary

Fig. 1, the mechano-sensor encapsulated with MY-133-V2000 has the highest sensitivity. The mechano-sensors encapsulated with polymer matrices with smaller or larger elastic moduli have lower force sensitivity.

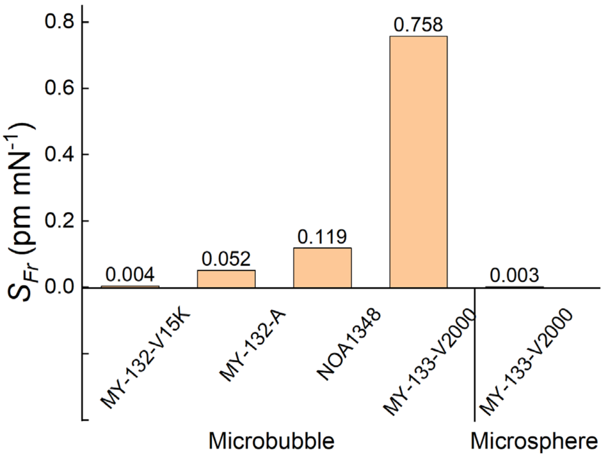

Supplementary Fig. 1. Experimental results of the force sensitivity comparison among microsphere-based mechano-sensor encapsulated with MY-133-V2000 and microbubble-based mechano-sensors encapsulated with four different polymer matrices (MY-132-V15K, MY-132-A, NOA1348, and MY-133-V2000).

The above experimental results indicate that the strain effect on the microbubble is the main reason for the shift in the resonance wavelength. Under the same external force, the strain effect on the solid microsphere is much weaker than that on the hollow microbubble. For the four microbubble-based mechano-sensors, the strain effect on the microbubble is not significant if the elastic modulus of the polymer matrix encapsulating the mechano-sensor is small, and the microbubble cannot deform if the elastic modulus of the polymer matrix encapsulating the mechano-sensor is too large. Therefore, the strain effect on the microbubble is strongest at an intermediate value of the elastic modulus.

Supplementary Table 1. Comparison of the properties of commercial UV-crosslinked low-refractive-index polymer.

Product name	RI at 950 nm	Elastic Modulus (MPa)	Poisson's Ratio	Hardness Shore
MY-132-A	1.322	0.4	0.495	30 A
MY-132-V15K	1.322	Very low	-	7
MY-133-V2000	1.329	5.2	0.41	70 A
NOA1348	1.348	158	-	30 D

Supplementary Note 3: Theoretical Analysis

The strain (ε_x, ε_y and ε_z) components along three coordinate axes in Cartesian coordinates are shown in Supplementary Fig. 2a. To simplify the mechano-sensor, the lever model is introduced (Supplementary Fig. 2b). The mechano-sensitive micro-hair serves as a lever in the system. The UV glue (Norland, NOA68) with an elastic modulus of 138 MPa and a hardness shore of 60 D is used for rigid fixation and serves as the fulcrum in the lever system. The low-refractive-index polymer matrix (MY-133-V2000) with an elastic modulus of 5.2 MPa and a hardness shore of 70 A is used to elastically fix and protect the microbubble and the fiber taper.

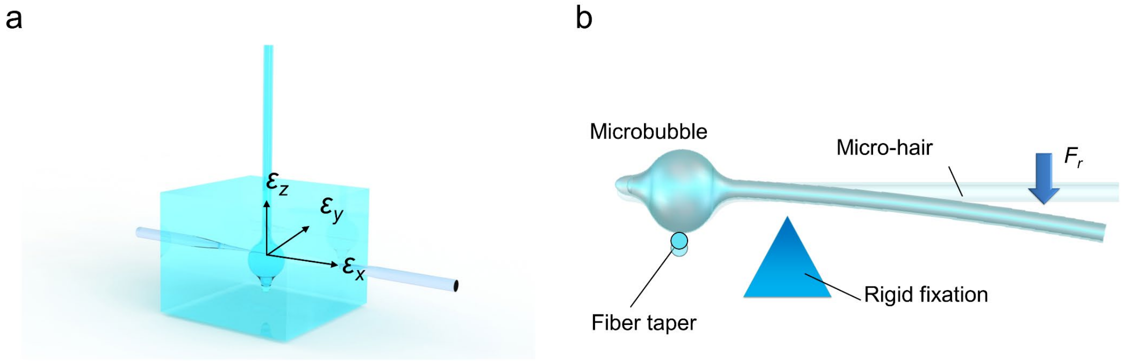

Supplementary Fig. 2. (**a**) Schematic illustration of the strain components (ε_x, ε_y and ε_z) along the three coordinate axes in Cartesian coordinates. (**b**) Schematic illustration of the mechano-sensor under an external force F_r, showing the design of the lever model.

A finite element method (FEM) model was used to analyze the responses of the mechano-sensor to applied external forces F_r. As a stimulus input in the simulation, an

external force F_r is applied in the $\varphi = 0°$ direction (Supplementary Fig. 3a), and the corresponding displacement is 2 μm. The stress field distribution in the equatorial cross-section of the mechano-sensor is shown in Supplementary Fig. 3b. The microbubble and fiber taper move in the $\varphi = 0°$ direction, and the stress effect of the polymer matrix is negligible. The radial strain dR/R and the strain-induced effective refractive index change dn_{eff}/n_{eff} in the equatorial cross-section of the microbubble are the averages of their field distribution integrals (Supplementary Fig. 3c and d). The radial strain field distribution along the microbubble's equatorial cross-section has a maximum negative value at $\varphi = 0°$ (that is, the same direction of F_r), a maximum positive value at $\varphi = 180°$ (that is, the opposite direction of F_r), and values of approximately zero at $\varphi = 90°$ and 270° (that is, the direction perpendicular to F_r). The fiber taper amplifies the radial strain at the coupling position (that is, $\varphi = 0°$ in the microbubble's equatorial cross-section), resulting in $dR/R < 0$ and a blueshift in the resonance wavelength.

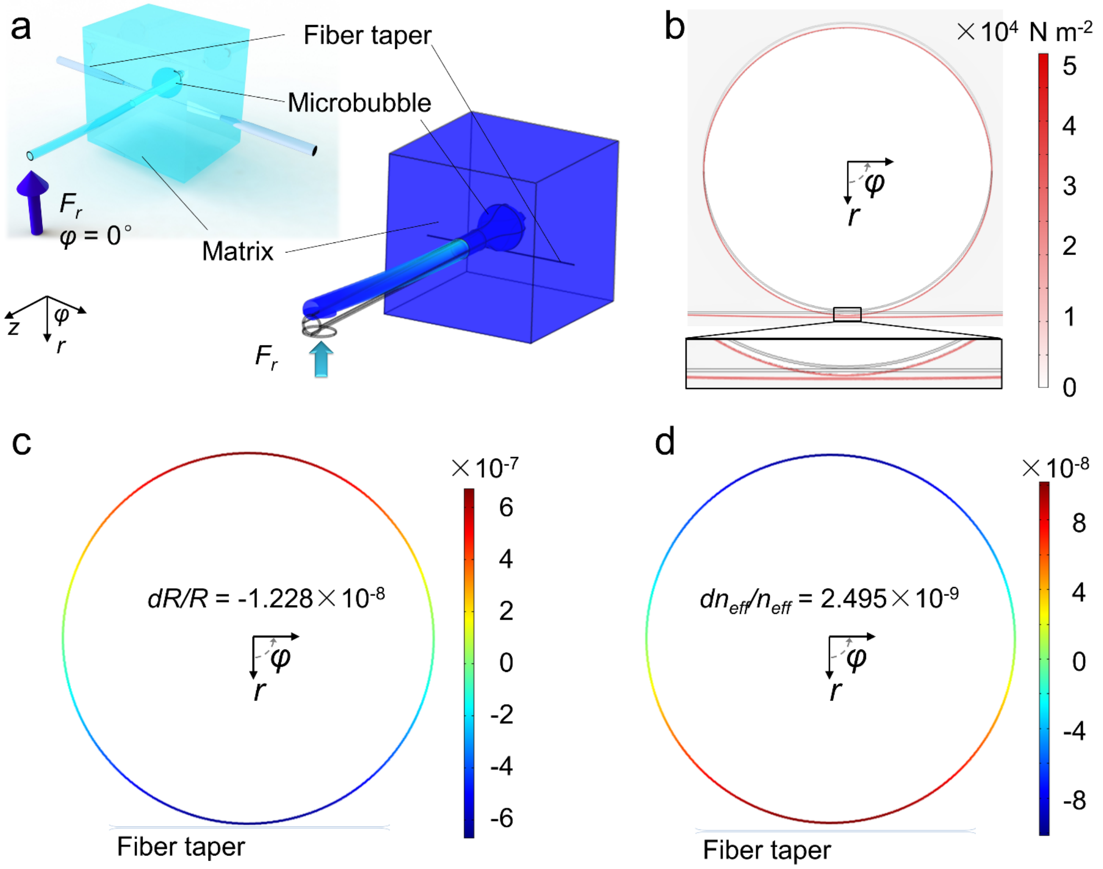

Supplementary Fig. 3. Simulations of the mechano-sensor under an external force F_r (φ = 0°). (**a**) Schematic illustration of the experimental device and FEM model of the mechano-sensor under an external force F_r (φ = 0°). (**b**) Stress field distribution in the r-φ plane under an external force F_r (φ = 0°). (**c**) and (**d**) Field distributions of the radial strain and strain-induced effective refractive index change along the equatorial cross-section of the microbubble under an external force F_r (φ = 0°), respectively.

Then, an external force F_r was applied in the φ = 90° direction (Supplementary Fig. 4a), and the stress field distribution in the equatorial cross-section of mechano-sensor is shown in Supplementary Fig. 4b. The microbubble and fiber taper move in the φ = 90° direction, and the stress effect of the polymer matrix is also negligible in this case. The field distributions of the radial strain and strain-induced effective refractive index change along the equatorial cross-section of the microbubble are shown in Supplementary Fig. 4c and d, respectively. The radial strain field distribution is negative in the range of φ = 0° to 180° in

the equatorial cross-section but positive in the range of φ = 180° to 360°. When the radial strain field integral distributed within the equatorial cross-section of the microbubble are averaged, dR/R and dn_{eff}/n_{eff} are both approximately zero. This is because the radial strain at the coupling position (that is, φ = 0° in the microbubble's equatorial cross-section) is approximately zero, resulting in a negligible strain amplification effect introduced by the fiber taper. There is no resonance wavelength shift when the external force F_r is applied in the φ = 90° or 270° directions.

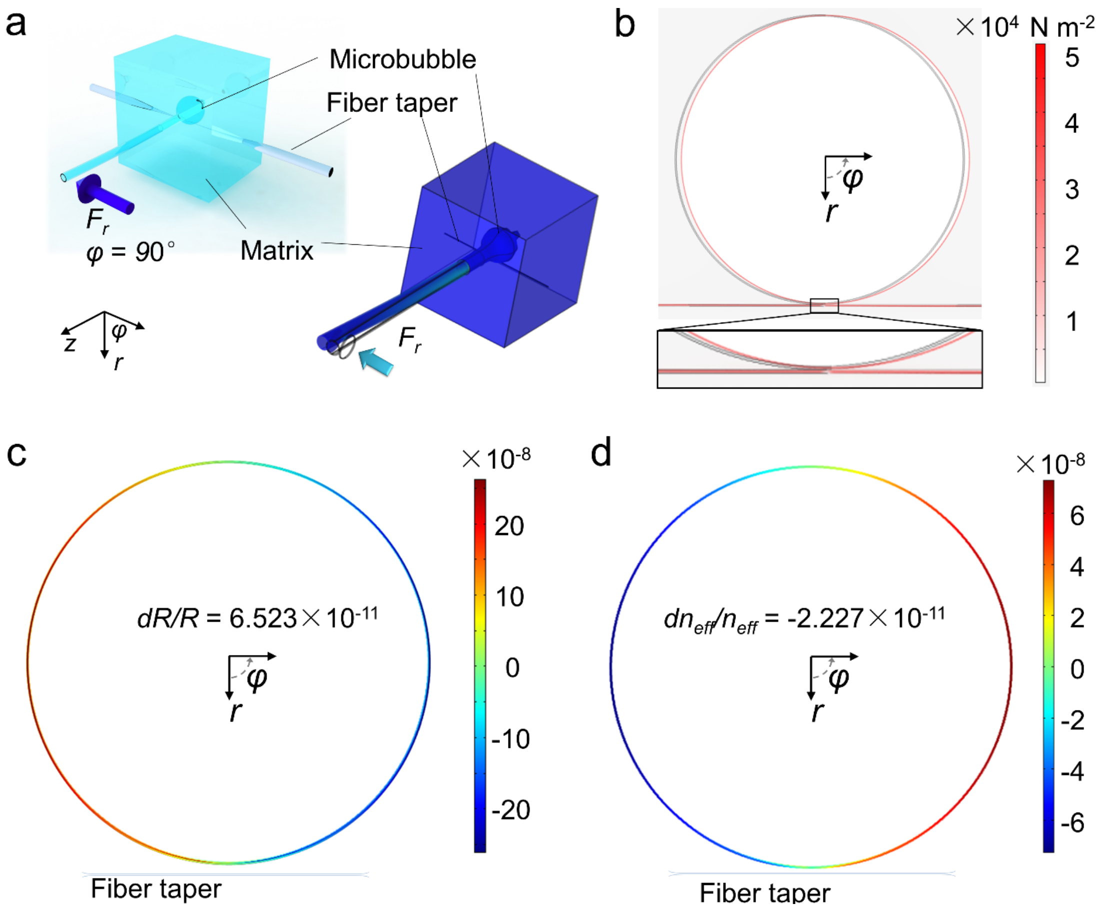

Supplementary Fig. 4. Simulations of the mechano-sensor under an external force F_r (φ = 90°). (**a**) Schematic illustration of the experimental device and FEM model of the mechano-sensor under an external force F_r (φ = 90°). (**b**) Stress field distribution in the r-φ plane under an external force F_r (φ = 90°). (**c**) and (**d**) Field distributions of the radial strain and strain-induced effective refractive index change along the equatorial cross-section of the microbubble under an external force F_r (φ = 90°), respectively.

Supplementary Note 4: Optimization of the Mechano-Sensor

To obtain a mechano-sensor with high displacement/force sensitivity, the geometric parameters of the micro-hair (that is, the glass capillary) and microbubble and the mechanical properties of the polymer matrix were optimized through the FEM scanning parameters. The outer contour of the microbubble can be approximately fitted with a Gaussian line shape:

$$r_{outer} = R_{capillary} + (R_{bubble} - R_{capillary}) \cdot e^{-\alpha z^2}. \tag{S1}$$

where $R_{capillary}$ is the radius of the capillary, R_{bubble} is the radius of the microbubble, and α is related to the expansion length of the microbubbles along the z-axis and is only determined by the discharge area of the fiber fusion splicer. Supplementary Fig. 5a shows a microbubble with a radius of 160 μm and a wall thickness of 1.5 μm prepared from a fused silica capillary with a radius of 61.5 μm and a corroded thickness of 10 μm. The outer contour of this microbubble is fitted with a Gaussian line shape (red line):

$$r_{outer} = 58.54 + 101.6 \cdot e^{-5.4\times10^{-5} z^2}. \tag{S2}$$

where 58.54 μm is basically consistent with the capillary outer radius of 61.5 μm, and 160.14 μm is basically consistent with the microbubble outer radius of 160 μm. When the hollow fused silica capillaries are used to fabricate the hollow microbubbles, the volume of the glass remains constant, so the curve for the inner contour of the microbubble can be formulated as:

$$r_{inner} = 48.54 + 110.1 \cdot e^{-5.598\times10^{-5} z^2}. \tag{S3}$$

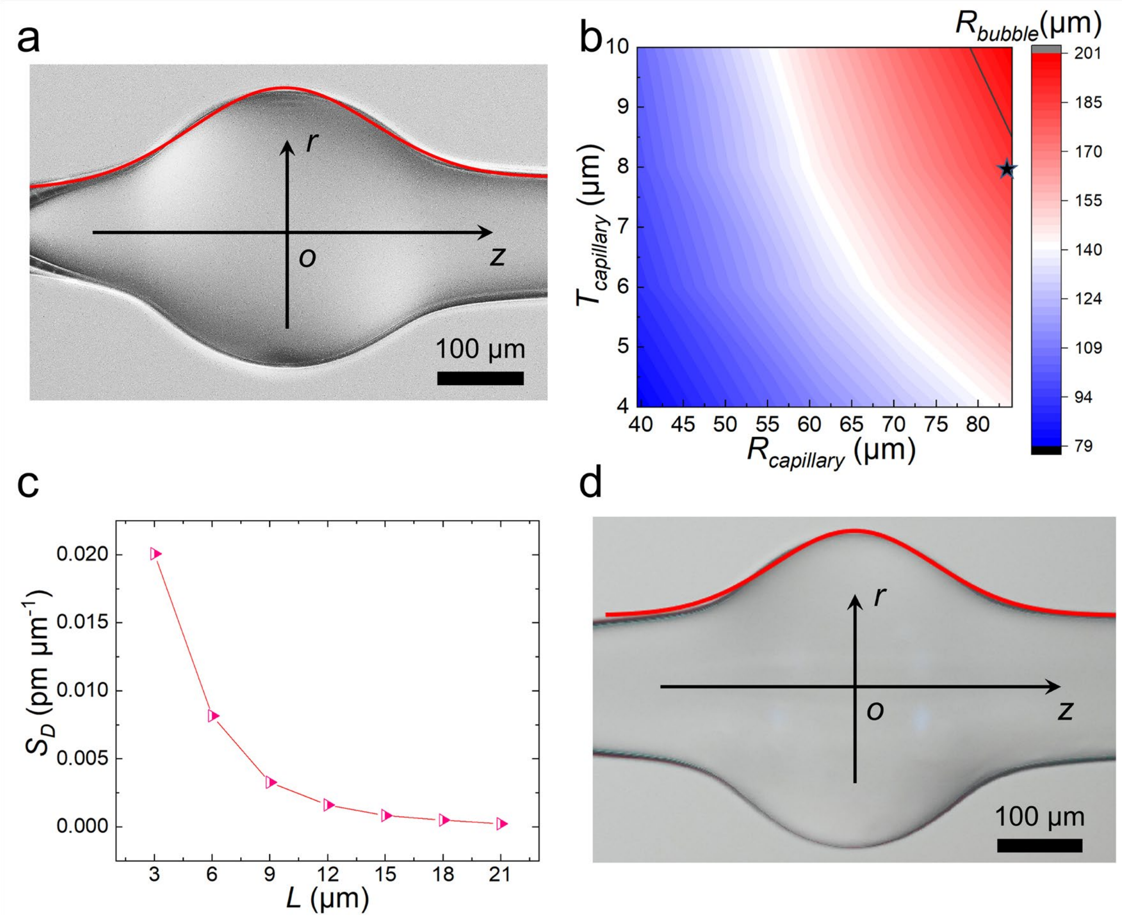

Supplementary Fig. 5. Parametric scanning of the geometric parameters of the capillary and microbubble. (**a**) The outer contour of a microbubble is fitted with a Gaussian line shape (red line). R_{bubble} and T_{bubble} are 160 μm and 1.5 μm, respectively. $R_{capillary}$ and $T_{capillary}$ are 61.5 μm and 10 μm, respectively. (**b**) Relations between R_{bubble} and the geometric parameters of the capillary (i.e., $R_{capillary}$ and $T_{capillary}$). T_{bubble} is fixed at 1.5 μm. The solid black line corresponds to the contour of R_{bubble} = 190 μm. The star label corresponds to the $R_{capillary}$ and $T_{capillary}$ selected for the micro-hair. (**c**) Simulations of the relations between the displacement sensitivity of the mechano-sensor (S_D) and the position of the applied force (L) under an external force F_r (φ = 180°). (**d**) Outer contour of the microbubble fabricated for mechano-opto-transduction is fitted with a Gaussian line shape (red line). R_{bubble} and T_{bubble} are 185 μm and 1.5 μm, respectively. $R_{capillary}$ and $T_{capillary}$ are 84 μm and 8 μm, respectively. The scale bars in (**a**) and (**d**) represent 100 μm.

In conclusion, the curves equation for the inner and outer contours of the microbubble produced by the fiber fusion splicer (FSU-975) can be formulated as:

$$\begin{aligned} r_{outer} &= R_{capillary} + (R_{bubble} - R_{capillary}) \cdot e^{-5.4\times10^{-5} z^2}, \\ r_{inner} &= (R_{capillary} - T_{capillary}) + (R_{bubble} - R_{capillary} + T_{capillary} - T_{bubble}) \cdot e^{-5.598\times10^{-5} z^2}. \end{aligned} \tag{S4}$$

where $T_{capillary}$ and T_{bubble} are the thicknesses of the capillary and microbubble walls, respectively. As shown in Supplementary Fig. 1, the strain effect on the microbubble is the main reason for the shift in the resonance wavelength, indicating that reducing T_{bubble} can effectively improve the displacement/force sensitivity of the mechano-sensor. In practice, considering that T_{bubble} is too thin to constrain the optical WGM and the stability of the preparation process, the thinnest T_{bubble} we can fabricate is 1.5 μm. Therefore, T_{bubble} is fixed at 1.5 μm in the following simulations and experiments.

Based on the principle of volume conservation during the preparation of the microbubbles and Equation (S4), once the geometric parameters of the capillary, including $R_{capillary}$ and $T_{capillary}$, are determined, the radius of the prepared microbubble (R_{bubble}) can be obtained (Supplementary Fig. 5b). $R_{capillary}$ and $T_{capillary}$ are determined to optimize the displacement sensitivity of the mechano-sensor (S_D). The FEM simulation results indicate that the larger the radius and wall thickness of the capillary are, the larger the radius of the prepared microbubble (Supplementary Fig. 5b), and ultimately, the higher the displacement sensitivity of the mechano-sensor (Fig. 2h). Because the circular symmetry of the prepared microbubble will be broken if R_{bubble} exceeds the limited discharge area of the fiber fusion splicer (FSU-975), R_{bubble} is limited to less than 190 μm (black solid line in Supplementary Fig. 5b and Fig. 2h). Finally, a fused silica capillary with a radius of 84 μm and a wall thickness of 8 μm is selected as the micro-hair (the star label in Supplementary Fig. 5b and Fig. 2h), and a microbubble with a radius of 185 μm and a wall thickness of 1.5 μm is fabricated for mechano-opto-transduction. In the FEM simulation, S_D varies with the action

point of the external force (L), as shown in Supplementary Fig. 5c. The outer contour curve of the prepared microbubble (Supplementary Fig. 5d) is fitted with a Gaussian line shape (red line):

$$r = 83.45 + 101.03 \cdot e^{-5.38\times10^{-5} z^2}. \tag{S5}$$

where 83.45 μm is basically consistent with the capillary outer radius of 84 μm, and 184.48 μm is basically consistent with the microbubble outer radius of 185 μm. This result indicates the rationality of the process for calculating the radius of the microbubble (Supplementary Fig. 5b and Equation (S4)).

The final version of the bioinspired optical mechano-sensor is shown in Supplementary Fig. 6, which is composed of a thin-walled glass microbubble integrated with a glass micro-hair that is optically coupled with a fiber taper at the equator of the microbubble resonator. To fit the outer contour curve of the microbubble with a Gaussian line shape and quantitatively control the geometric parameters of the microbubble, the prepared microbubble is approximately 200 μm away from the melting node where the glass capillary is sealed. Notably, such a short distance of 200 μm does not affect the displacement sensitivity of the mechano-sensor. The radius ($R_{capillary}$) and wall thickness ($T_{capillary}$) of the glass capillary are 84 μm and 8 μm, respectively. The radius (R_{bubble}) and wall thickness (T_{bubble}) of the glass-based hollow microbubble are 185 μm and 1.5 μm, respectively. The 50 mm long micro-hair collects information about the mechanical stimuli in the environment, such as forces and vibrations, and propagates the information to the microbubble. The thin-walled microbubble configuration is mechanically flexible, allowing the device to transform these environmental mechanical stimuli into shifts in the WGM resonance wavelength and the spectral light signals (i.e., shifts in the dips).

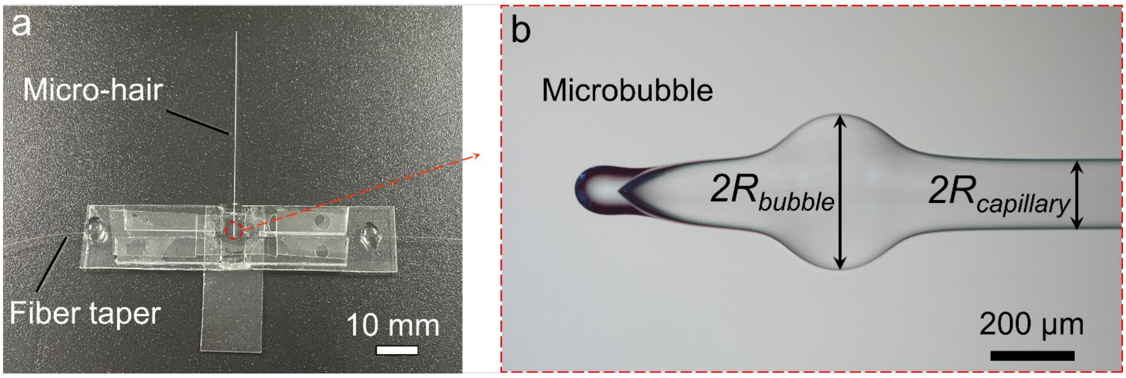

Supplementary Fig. 6. (**a**) Image of the mechano-sensor. The scale bar represents 10 mm. (**b**) Optical microscope image of a microbubble integrated with a glass micro-hair standing on its top center. The scale bar represents 200 μm.

As shown in Supplementary Fig. 14, prior to the preparation of a microbubble from a fused silica capillary, the coating on the capillary surface needs to be removed. The coating can be removed from the entire capillary surface or only selectively from the microbubble surface. To analyze the effect of the coating removal ratio on the displacement sensitivity, an FEM model was used. As shown in Supplementary Fig. 7, when the capillary has no coating in part of the polymer matrix, the proportion of coating has little effect on the displacement sensitivity of the mechano-sensor. However, when the proportion of coating present is large enough that the capillary is coated in the part of the polymer matrix, the displacement sensitivity is greatly reduced. Therefore, it is necessary to ensure that the capillary in the polymer matrix is not coated after encapsulation.

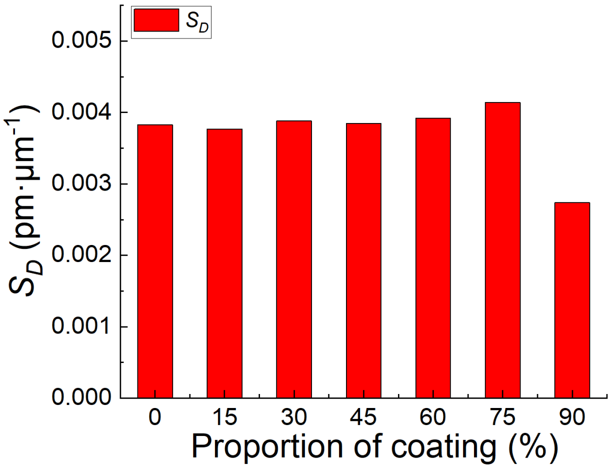

Supplementary Fig. 7. Effect of the coating removal ratio on the displacement sensitivity of mechano-sensor.

Finally, the mechanical properties of the polymer matrix, including the elastic modulus E and Poisson's ratio υ, were optimized with fixed geometric parameters of micro-hair and microbubble. As shown in Fig. 2i, when the elastic modulus of the polymer matrix is 5 MPa, the strain effect is the strongest, and the displacement sensitivity of the mechano-sensor is the highest. An increase or decrease in the magnitude of the elastic modulus will reduce the displacement sensitivity of the mechano-sensor, which corresponds to the experimental results in Supplementary Fig. 1. The reason may be that the strain effect on the microbubble is not significant if the elastic modulus of the polymer matrix is too small, and the microbubble can not be deformed if the elastic modulus of the polymer matrix is too large. Poisson's ratio has relatively little influence on the displacement sensitivity. Thus, considering the commercial UV-crosslinked low-refractive-index polymers data (Supplementary Table 1), the MY-133-V2000 polymer with an elastic modulus E of 5.2 MPa and Poisson's ratio υ of 0.41 is selected as the polymer matrix.

Supplementary Note 5: Measurement Schematic Diagram of External Force F_r

The full width at half maximum (FWHM) varies intricately as the external force F_r (φ= 180°) increases from 0.12 mN to 0.72 mN in steps of 0.12 mN (Supplementary Fig. 8a), which can be attributed to complex influence factors (i.e., the strain effect on the microbubble, the scattering loss caused by the gradual strain-induced effective refractive index change, and the change in the gap between the microbubble and the fiber taper). The theoretical calculation formula of the spring constant of the mechano-sensor can be expressed as:

$$k = \frac{F_r}{L}. \tag{S6}$$

Here, k (N m^{-1}) is the spring constant of the mechano-sensor, F_r (N) is the external force loaded at the action point of the micro-hair, and L (m) is the position of the force action point. The spring constant of the mechano-sensor (k) varies from 61.861 N m^{-1} to 0.412 N m^{-1} as L increases from 3 mm to 15 mm (Supplementary Fig. 8b).

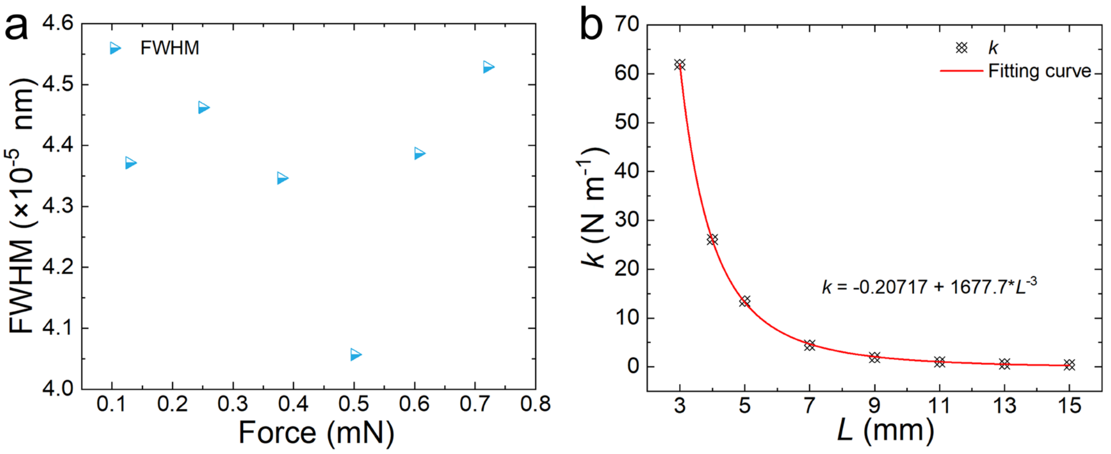

Supplementary Fig. 8. (**a**) Full width at half maximum (FWHM) varies intricately as the external force F_r increases from 0.12 mN to 0.72 mN. (**b**) Relations between the spring constant of the mechano-sensor (k) and the position of the force action point (L). k is inversely proportional to the third power of L.

To measure the directional characteristics of the mechano-sensor with external force (F_r), the mechano-sensor is mounted on a rotating device, which drives the mechano-sensor to rotate 360° around the z-axis. The displacement and force sensitivities of the mechano-sensor are measured at 10° intervals, and the directional diagram of the mechano-sensor is finally obtained (Supplementary Fig. 9). The static force value applied to the mechano-sensor micro-hair is given by a commercial force sensor. The commercial force sensor is mounted on a 3D stepper motor stage and equipped with a digital force gauge. The object applying the external force is driven by the stepper motor, and the wavelength shift and the static force change are recorded by a computer and a digital force gauge in real time, respectively. The

displacement value is obtained by the stepper motor. The inset shows the position of the force action point (L).

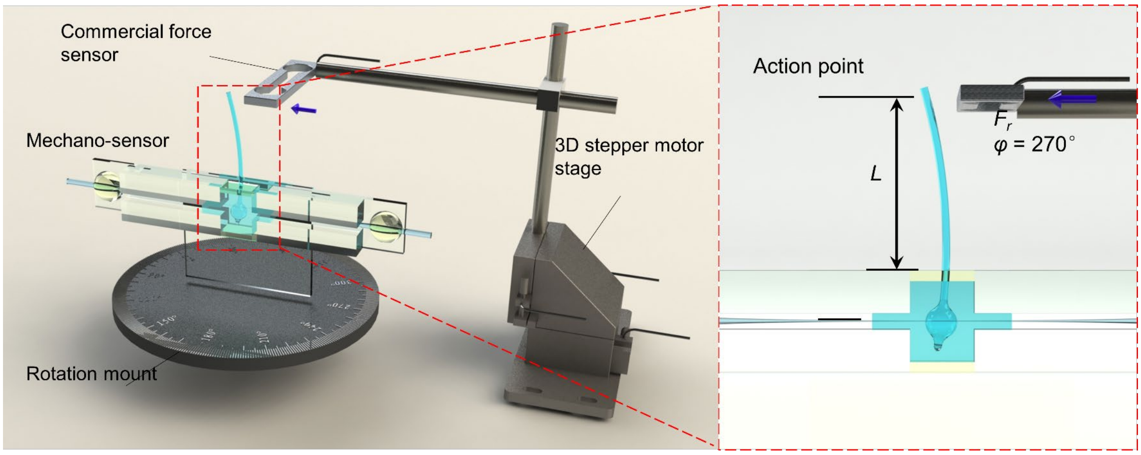

Supplementary Fig. 9. Measurement schematic diagram of the external force (F_r) applied to the mechano-sensor micro-hair in the r-φ plane. Inset: Front view of the mechano-sensor.

Supplementary Note 6: Anti-Interference Capability Measurement

When perceiving mechanical stimuli in the environment, the mechano-sensor may be disturbed by temperature changes in the external environment. Moreover, if working in acidic, alkaline, or salt environments, such as seawater, the electrical mechano-sensor may be corroded.

To demonstrate the anti-interference capability (i.e., temperature interference) of the mechano-sensor, temperature is introduced as a variable during the displacement measurement[10]. To construct the two-dimensional (2D) sensing matrix $M_{T,D}$, the sensitivities of two WGMs are measured to decouple the temperature and displacement measurements. The 2D sensitivity matrix $M_{T,D}$ is defined as:

$$M_{T,D} = \begin{bmatrix} S_{T1} & S_{D1} \\ S_{T2} & S_{D2} \end{bmatrix}. \tag{S7}$$

Here, S_T and S_D are the temperature and displacement sensitivities, respectively. The wavelength shifts of the two WGMs ($\Delta\lambda_1$ and $\Delta\lambda_2$) induced by temperature (ΔT) and displacement changes (ΔD) are defined as:

$$\begin{bmatrix} \Delta\lambda_1 \\ \Delta\lambda_2 \end{bmatrix} = M_{T,D} \begin{bmatrix} \Delta T \\ \Delta D \end{bmatrix}. \tag{S8}$$

Therefore, the changes in temperature and displacement can be solved by the following matrix:

$$\begin{bmatrix} \Delta T \\ \Delta D \end{bmatrix} = M_{T,D}^{-1} \begin{bmatrix} \Delta\lambda_1 \\ \Delta\lambda_2 \end{bmatrix}. \tag{S9}$$

First, Supplementary Fig. 10a shows the evolution of the transmission spectra as the temperature increases from 27.7°C to 28.2°C at 0.1°C intervals, while the displacement is kept at 0 μm. The temperature sensitivities (S_{T1} and S_{T2}) of the two tracked WGMs are -16.495 pm °C^{-1} and -21.665 pm °C^{-1}, respectively (Supplementary Fig. 10b). The greater negative thermo-optical effect of the polymer matrix and the weaker positive thermo-optical effect of the glass wall both lead to the blueshift in the resonance wavelength with increasing temperature.

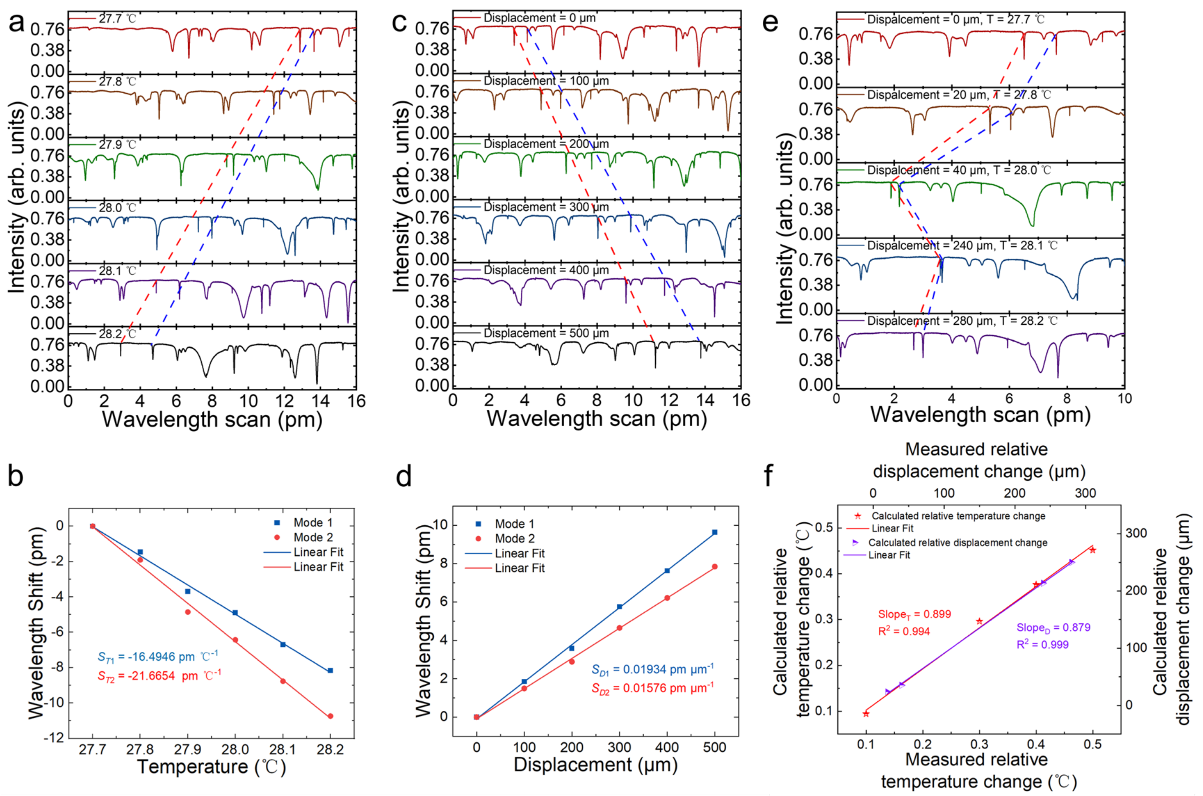

Supplementary Fig. 10. (**a**) Transmission spectra evolution of the mechano-sensor as the temperature increases from 27.7°C to 28.2°C. arb. units, arbitrary units. (**b**) The temperature sensitivities of two tracked WGMs. (**c**) Transmission spectra evolution of the mechano-sensor as the displacement increases from 0 μm to 500 μm. arb. units, arbitrary units. (**d**) The displacement sensitivities of two tracked WGMs. (**e**) Transmission spectra evolution of the mechano-sensor at different temperatures and displacements (0 μm and 27.7°C, 20 μm and 27.8°C, 40 μm and 28.0°C, 240 μm and 28.1°C, and 280 μm and 28.2°C). arb. units, arbitrary units. (**f**) Temperature/displacement comparisons between the calculated and measured results.

Second, Supplementary Fig. 10c shows the evolution of the transmission spectra as the displacement increases from 0 μm to 500 μm at 100 μm intervals (L = 5 mm, φ = 180°), while the temperature is kept at 27.7°C. The displacement sensitivities (S_{D1} and S_{D2}) of the two above tracked WGMs are 0.0193 pm μm^{-1} and 0.0158 pm μm^{-1} (Supplementary Fig. 10d).

Because different WGMs have various energy ratios in the polymer matrix and glass wall, mode 1 and mode 2 have distinct temperature and displacement sensitivities. Mode 2 has more energy leakage than mode 1 in the polymer matrix, so mode 2 has a larger temperature sensitivity. However, mode 2 has a lower energy proportion in the glass wall with the largest strain effect, so the displacement sensitivity of mode 2 is smaller.

Finally, the evolution of the transmission spectra as the temperature and displacement changed concurrently was demonstrated (Supplementary Fig. 10e). By tracking the two above tracked WGMs and applying Equation S9, the displacement and temperature measurements can be decoupled, as shown in Supplementary Fig. 10f. The derived root mean square errors of the displacement and temperature are 20.15 μm and 0.027°C, respectively. The results show that the mechano-sensor has good displacement-temperature decoupling stability. Moreover, compared to electrical mechano-sensors, the optical mechano-sensor has fascinating features, such as all-optical multifunctional perception system.

Moreover, to demonstrate that the mechano-sensor can work properly in saline and alkaline environments, the mechano-sensor was immersed in sea water. The sea water was prepared according to ASTM standard D1141-98 (2013, American Society for Testing Materials)[11]. An external force F_r (φ = 0°, L = 12 mm) is applied on the micro-hair, with the displacement increasing from 0 μm to 90 μm in steps of 30 μm (Supplementary Fig. 11a). The calculated displacement sensitivity is -0.00255 pm μm^{-1} (Supplementary Fig. 11b). The result is comparable to that in Fig. 3e. Therefore, the force sensitivity is -3.131 pm mN^{-1} according to Fig. 3e, and the calculated external force F_r is shown in Supplementary Fig. 11c.

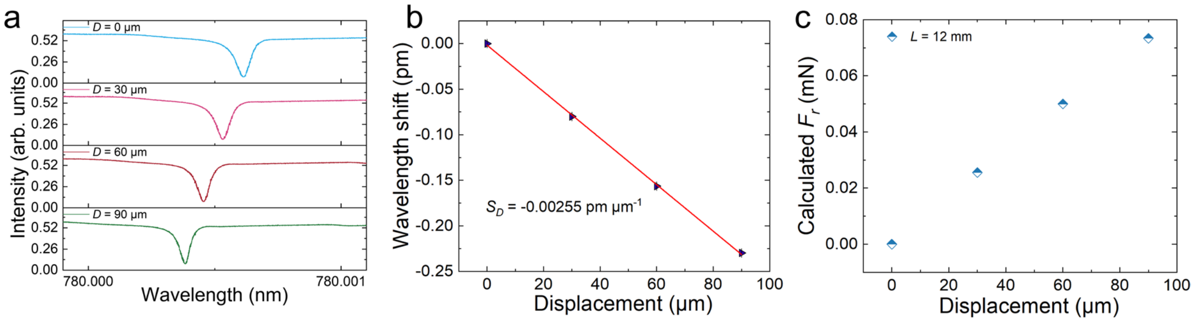

Supplementary Fig. 11. (**a**) Transmission spectra evolution of the mechano-sensor as the displacement increases from 0 μm to 90 μm in the sea water environment. arb. units, arbitrary units. (**b**) Displacement sensitivity of the mechano-sensor immersed in sea water. (**c**) Relation between the calculated F_r and the displacement.

Supplementary Note 7: Measurement Schematic Diagram of the External Force F_z

As shown in the measurement schematic diagram in Supplementary Fig. 12, the external force (F_z) applied along the *z*-axis of the mechano-sensor is the same as that shown in Supplementary Fig. 8, except that the direction of the applied force is changed.

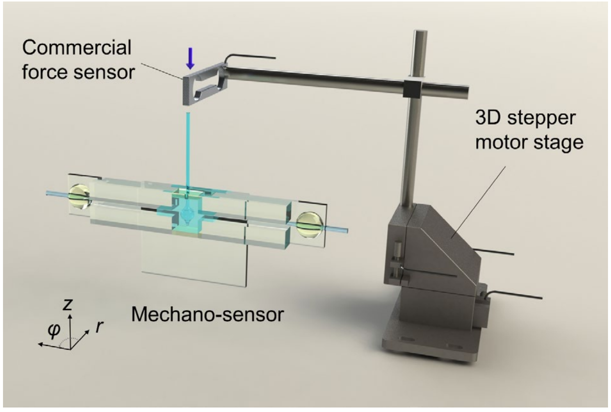

Supplementary Fig. 12. Measurement schematic diagram of the external force (F_z).

Supplementary Note 8: Durability Test of the Mechano-Sensor

There are two methods to investigate the durability and response of the mechano-sensor: tracking the resonance wavelength shift of the WGM ($\Delta\lambda$) and monitoring the intensity change in the WGM (ΔI).

When tracking $\Delta\lambda$ (Fig. 5d and Supplementary Fig. 13b), the output wavelength of the tunable laser is tuned by an external triangular wave, covering the WGM spectrum to extract the resonance wavelength. The response time and recovery time of the frequency response by applying a square waveform with a frequency of 0.8 Hz to the piezo actuator are calculated as 18 ms and 20 ms, respectively (Supplementary Fig. 13c). The mechano-sensor can detect a square waveform with a frequency up to 16 Hz, as demonstrated in Supplementary Fig. 13b. However, it is inaccurate to measure the response time by tracking $\Delta\lambda$. Due to the limitation of the maximum scanning frequency of the external triangular wave (below 40 Hz), the minimum time interval between data points in Supplementary Fig. 13c is 25 ms, exceeding the calculated response time of 18 ms. Therefore, tracking $\Delta\lambda$ can not accurately capture the response time, which is likely less than 18 ms.

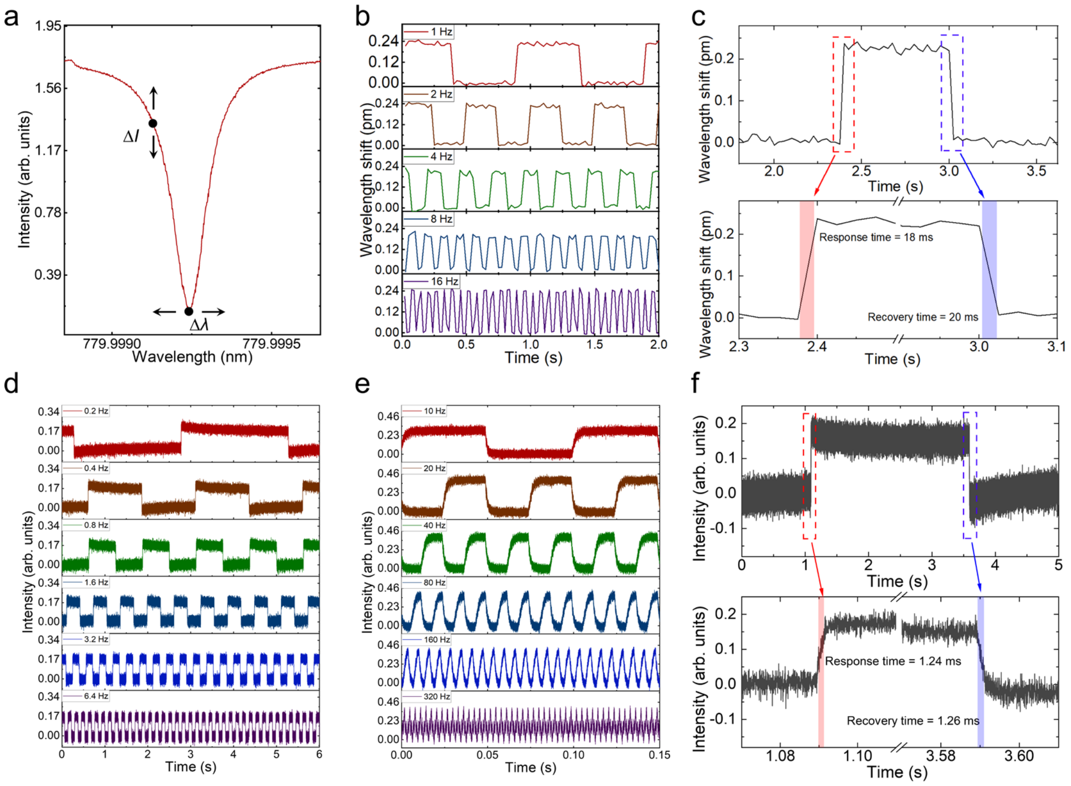

Supplementary Fig. 13. (**a**) Schematic diagram for tracking the wavelength shift ($\Delta\lambda$) of the WGM and changes in the WGM intensity (ΔI). arb. units, arbitrary units. (**b**) Higher frequency responses of the mechano-sensor by tracking the wavelength shifts. Square signals are applied to the piezo actuator at frequencies of 1 Hz, 2 Hz, 4 Hz, 8 Hz, and 16 Hz, respectively. (**c**) Response time and recovery time of the mechano-sensor, determined by tracking the wavelength shifts. (**d**) Low frequency responses of the mechano-sensor by monitoring the intensity changes. Square signals are applied to the piezo actuator at frequencies of 0.2 Hz, 0.4 Hz, 0.8 Hz, 1.6 Hz, 3.2 Hz, and 6.4 Hz. arb. units, arbitrary units. (**e**) Higher frequency responses of the mechano-sensor by monitoring the intensity changes. Square signals are applied to the piezo actuator at frequencies of 10 Hz, 20 Hz, 40 Hz, 80 Hz, 160 Hz, and 320 Hz, respectively. arb. units, arbitrary units. (**f**) Response time and recovery time of the mechano-sensor, determined by monitoring the intensity change in the WGM. arb. units, arbitrary units.

In contrast, when monitoring ΔI (Supplementary Fig. 13a), there is no need to scan the entire WGM spectrum. Instead, the output wavelength remains at the rising or falling edge of the resonance dip, and the response time of the mechano-sensor can be accurately obtained by monitoring ΔI through the oscilloscope. The accurate response and recovery times of 1.24 ms and 1.26 ms, respectively, are obtained by applying a square waveform with a frequency of 0.2 Hz to the piezo actuator (Supplementary Fig. 13f). The mechano-sensor can detect a square waveform with a frequency up to 320 Hz, as demonstrated in Supplementary Fig. 13d and e.

Notably, while monitoring ΔI can accurately capture the response time of the mechano-sensor, this method is strongly dependent on the selection of the start position and the nonlinearity change in the intensity. Thus, it is more reasonable for us to investigate the perception capability of the mechano-sensor using the wavelength shift approach.

Supplementary Table 2. Comparison of the sensing performance of different types of force sensors.

Structures	Platform/Material	Response time/ms	Recovery time/ms	Sensitivity	*DL*	Refs.
Dual-modal piezotronic transistor	ZnO nano/microwire	360	360	221.5 N^{-1}	21 mN	[12]
Electrospun micropyramid arrays on-skin devices	Poly(vinylidene fluoride) film	0.8	-	19 kPa^{-1}	0.05 Pa (13 mN)	[13]
Electronic whiskers	Shape memory polymer and gold strain gauges [14]<br>Pizeoresistor [15]<br>MEMS barometers [16]<br>Graphite pencil trace [17]<br>CNT-Ag NP film [18,19]<br>Graphene [20]<br>Fluorinated ethylene propylene [21]<br>Giant magnetoresistive sensor [22,23]	0.25 [14]<br>16 [15]<br>37 [16]<br>50 [17]<br>90 [18]<br>100 [19]<br>220 [20]	65 [15]<br>76 [17]	46 Ω mN^{-1} [23]<br>80 kPa^{-1} [19]	1.129 μN [21]<br>3.33 μN [16]<br>632 μN [22]<br>1.31 mN [23]	[14-23]
Photonic whisker	Silica microbubble	1.24	1.26	3.994 pm mN^{-1}	0.9 μN	This work

Supplementary Note 9: Fabrication Procedures for the Mechano-Sensor

The fabrication of the microbubble is shown in Supplementary Fig. 14. Initially, to prepare a thin-walled hollow microbubble, a fused silica capillary (Zhengzhou INNOSEP Scientific, China) needed to be corroded by hydrofluoric acid until the corroded wall thickness was 4~10 μm. Subsequently, a section of a silica capillary was placed at the electrodes of an optical fiber fusion splicer. When the electrodes were discharged, one end of the silica capillary was sealed. Then, the outer coating layer of the silica capillary was burnt with an alcohol burner and wiped with microscope lens paper. The silica capillary was placed at the center of the electrodes. The other end of the silica capillary was connected to a Teflon tube and a syringe. While the syringe was being pushed and the electrodes were discharging, the

silica capillary was heated, melted and expanded, and finally, a hollow core microbubble structure with a long mechano-sensor micro-hair was fabricated.

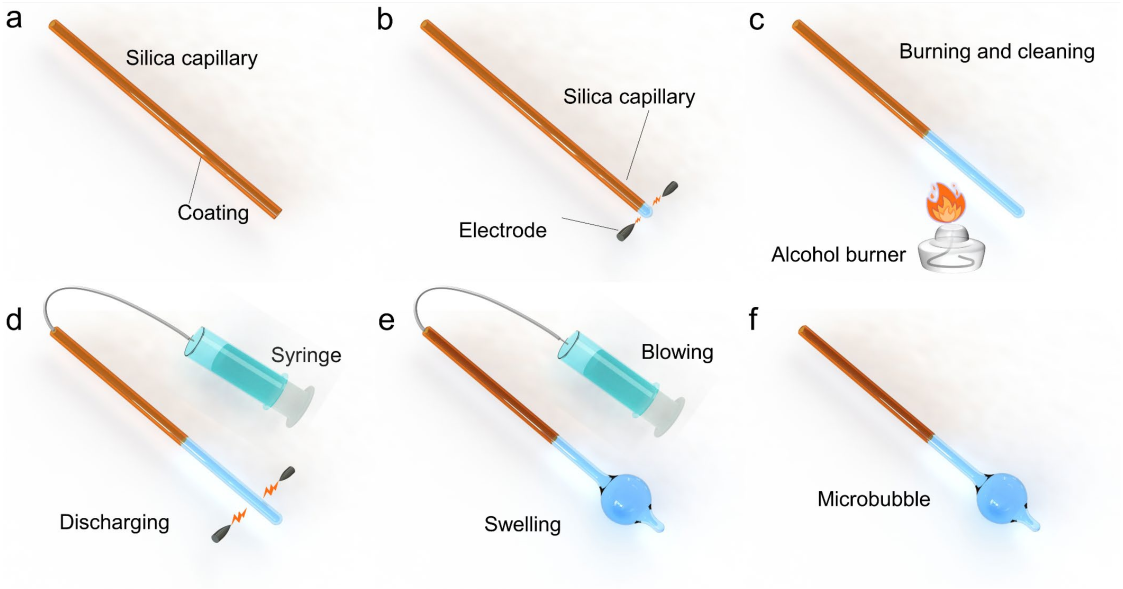

Supplementary Fig. 14. Fabrication procedures for the microbubble resonator. (**a**) A silica capillary was used, and (**b**) the end of the capillary was sealed by electrode discharge. (**c**) The coating was burned, and the capillary was cleaned with microscope lens paper. (**d**) The capillary was connected to a syringe and placed under the electrodes. (**e**) The electrodes were discharged, and air was blown into the capillary, causing it to swell. (**f**) Finally, the prepared microbubble resonator with a long mechano-sensor micro-hair was obtained.

The fabrication procedures for the bioinspired mechano-sensor are shown in Supplementary Fig. 15. The UV glue (Norland, NOA68) with an elastic modulus of 138 MPa and a hardness shore of 60 D was used for rigid fixation and a hard clamp. The polymer matrix (MY-133-V2000) with an elastic modulus of 5.2 MPa and a hardness shore of 70 A was used to elastically fix and protect the microbubble and the fiber taper. First, the fiber taper was fixed on a glass scaffold with NOA68 glue (Supplementary Fig. 15a–c). Second, the gap between the fiber taper and glass scaffold was filled with the polymer matrix (Supplementary Fig. 15d). Next, the microbubble was accurately controlled by five-dimensional optic alignment stages to realize the best coupling condition with the fiber taper

(Supplementary Fig. 15e), and the micro-hair was glued on the glass scaffold by NOA68 glue. Afterward, the optical coupling region between the fiber taper and the microbubble was completely wrapped with the polymer matrix to improve the stability of the sensor (Supplementary Fig. 15f).

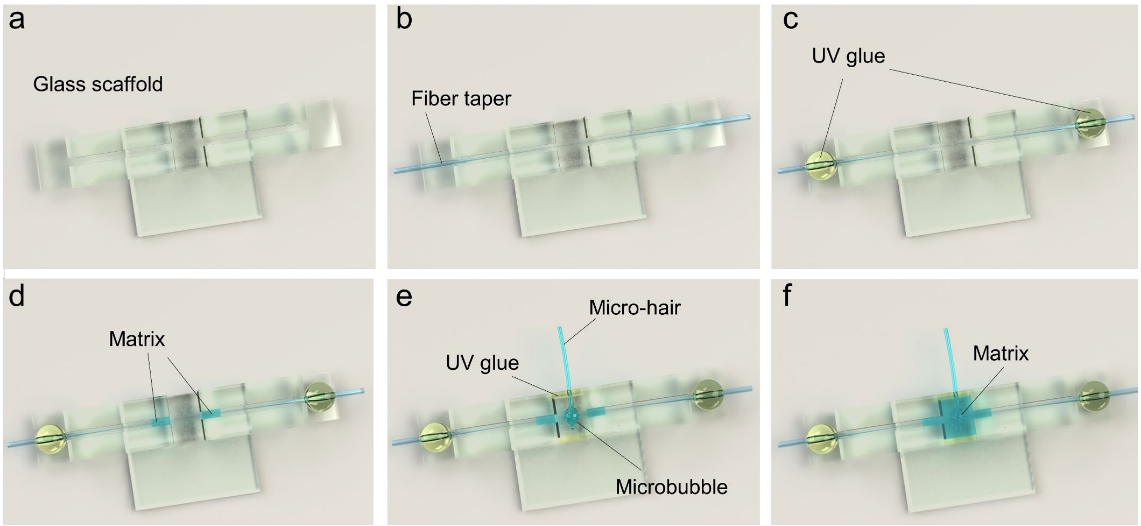

Supplementary Fig. 15. Fabrication procedures for the bioinspired mechano-sensor. (**a**) A glass scaffold was used for microbubble-fiber taper coupling. (**b**, **c**) Fiber taper was placed on the glass scaffold and fixed with UV glue. (**d**) Gap between the fiber taper and glass scaffold was filled with matrix. (**e**) Microbubble with micro-hair was coupling with the fiber taper and fixed. (**f**) Optical coupling region was wrapped with matrix.

Supplementary References

1. Marcotti, W. Functional assembly of mammalian cochlear hair cells. *Exp. Physiol.* **97,** 438-451 (2012).
2. Fratzl, P. & Barth, F. G. Biomaterial systems for mechanosensing and actuation. *Nature* **462,** 442-448 (2009).
3. McHuron, E. A., Holser, R. R. & Costa, D. P. What's in a whisker? Disentangling ecological and physiological isotopic signals. *Rapid Commun. Mass Spectrom.* **33,** 57-66 (2018).

4. Dijkstra, M. et al. Artificial sensory hairs based on the flow sensitive receptor hairs of crickets. *J. Micromech. Microeng.* **15,** S132-S138 (2005).

5. Alfadhel, A. & Kosel, J. Magnetic nanocomposite cilia tactile sensor. *Adv. Mater.* **27,** 7888-7892 (2015).

6. Tao, J. & Yu, X. Hair flow sensors: from bio-inspiration to bio-mimicking—a review. *Smart Mater. Struct.* **21,** 113001 (2012).

7. Ma, E. Y. & Raible, D. W. Signaling pathways regulating zebrafish lateral line development. *Curr. Biol.* **19,** R381-R386 (2009).

8. McConney, M. E., Anderson, K. D., Brott, L. L., Naik, R. R. & Tsukruk, V. V. Bioinspired material approaches to sensing. *Adv. Funct. Mater.* **19,** 2527-2544 (2009).

9. Albert, J., Friedrich, O., Dechant, H. E. & Barth, F. Arthropod touch reception: spider hair sensilla as rapid touch detectors. *J. Comp. Physiol. A Sens. Neural Behav. Physiol.* **187,** 303-312 (2001).

10. Wu, Y. et al. Simultaneous temperature and pressure sensing based on a single optical resonator. *Opt. Express* **31,** 18851-18861 (2023).

11. Wang, H., Gao, M., Guo, Y., Yang, Y. & Hu, R. A natural extract of tobacco rob as scale and corrosion inhibitor in artificial seawater. *Desalination* **398,** 198-207 (2016).

12. Ge, R., Yu, Q., Zhou, F., Liu, S. & Qin, Y. Dual-modal piezotronic transistor for highly sensitive vertical force sensing and lateral strain sensing. *Nat. Commun.* **14,** 6315 (2023).

13. Zhang, J. et. al. Versatile self-assembled electrospun micropyramid arrays for high-performance onskin devices with minimal sensory interference. *Nat. Commun.* **13,** 5839 (2022).

14. Reeder, Jonathan T. et al. 3D, reconfigurable, multimodal electronic whiskers via directed air assembly. *Adv. Mater.* **30,** 1706733 (2018).

15. Wang, Q. et al. Mechano-Sensor for proprioception inspired by ultrasensitive trigger hairs of Venus flytrap. *Cyborg Bionic Syst.*

16. Deer, W., & Pounds, P. E. Lightweight whiskers for contact, pre-contact, and fluid velocity sensing. *IEEE Robot Autom Let.* **4,** 1978-1984 (2019).

17. Hua, Q. et al. Bioinspired Electronic Whisker Arrays by Pencil-Drawn Paper for Adaptive Tactile Sensing. *Adv. Electron. Mater.* **2,** 1600093 (2016).

18. Harada, S. et al. Fully printed, highly sensitive multifunctional artificial electronic whisker arrays integrated with strain and temperature sensors. *ACS nano* **8,** 3921-3927 (2014).

19. Takei, K. et al. Highly sensitive electronic whiskers based on patterned carbon nanotube and silver nanoparticle composite films. *Proc. Natl. Acad. Sci. U. S. A.* **111,** 1703-1707 (2014).

20. Gul, J. Z. et al. Fully 3D printed multi-material soft bio-inspired whisker sensor for underwater-induced vortex detection. *Soft Robot.* **5,** 122-132 (2018).

21. An, J. et al. Biomimetic hairy whiskers for robotic skin tactility. *Adv. Mater.* **33,** 2101891 (2021).

22. Ribeiro, P. et. al. A miniaturized force sensor based on hair-like flexible magnetized cylinders deposited over a giant magnetoresistive sensor. *IEEE Trans. Magn.* **53,** 1-5 (2017).

23. Alfadhel, A. et. al. A magnetoresistive tactile sensor for harsh environment applications. *Sensors* **16,** 650 (2016).
