# Supplementary material for: An all-optical multidirectional mechano-sensor inspired by biologically mechano-sensitive hair sensilla
Source: Nat Commun. 2024 Apr 4;15:2906. doi: 10.1038/s41467-024-47299-0 (PMC10994919; doi:10.1038/s41467-024-47299-0)
Supplement: Supplementary file 3 — Description of Additional Supplementary Files [file 41467_2024_47299_MOESM3_ESM.docx]

**Description of Additional Supplementary Files**

**Supplementary Video 1:**

Responses of the quadruped cat robot equipped with the mechano-sensor system
